# From linear models to deep learning: statistical advances in genomic selection for animal breeding

**DOI:** 10.1186/s40104-026-01477-w

**Published:** 2026-08-07

**Authors:** Lifei Zhang, Mingzhu Zhang, Xinle Wang, Cancan Chen, Shunzhe Wang, Yujie Zhao, Yanjun Zhang, Ruijun Wang, Yongbin Liu

**Affiliations:** College of Animal Science, Inner Mongolia Agricultural University, Hohhot, 010018 China

**Keywords:** Animal breeding, Bayesian, Deep learning, GBLUP, GEBV, Genomic selection, Machine learning, Prediction accuracy, ssGBLUP

## Abstract

Genomic selection (GS) has revolutionized animal breeding by accelerating genetic gain through genome-wide marker data. As genotyping technologies advance and data dimensionality grows, the statistical foundations of GS are shifting from classical linear frameworks, which assume additive genetic effects, toward advanced computational models that capture complex nonlinear relationships in genomic data. The commercialization of genotyping arrays for livestock and poultry, coupled with steadily declining sequencing costs, has led to an exponential increase in the availability of high-density genomic data. However, challenges persist, including scenarios where the number of genetic markers far exceeds the number of samples with phenotypic data, and the growing complexity of relationships within genomic data. These issues significantly limit the applicability of traditional evaluation models. In parallel, computational power has increased significantly over the last few decades, providing the capacity necessary for highly complex analyses. While traditional linear mixed models provide a robust framework for incorporating biological priors and modeling additive genetic effects, they often rely on simplified assumptions. In contrast, machine learning (ML) and deep learning (DL) algorithms, which do not rely on predefined parametric models, are well-suited to capturing complex nonlinear relationships and offer effective solutions to the aforementioned challenges. This review provides a comprehensive overview of GS methodologies. We first cover the statistical foundations of linear mixed and Bayesian models, and then survey modern ML and DL approaches. We discuss the assumptions, advantages, and limitations of each method and, by comparing the computational efficiency and predictive accuracy of these diverse approaches, aim to provide practical guidance for optimizing genomic evaluation strategies in the era of big data breeding.

## Introduction

**Fig. 1:**
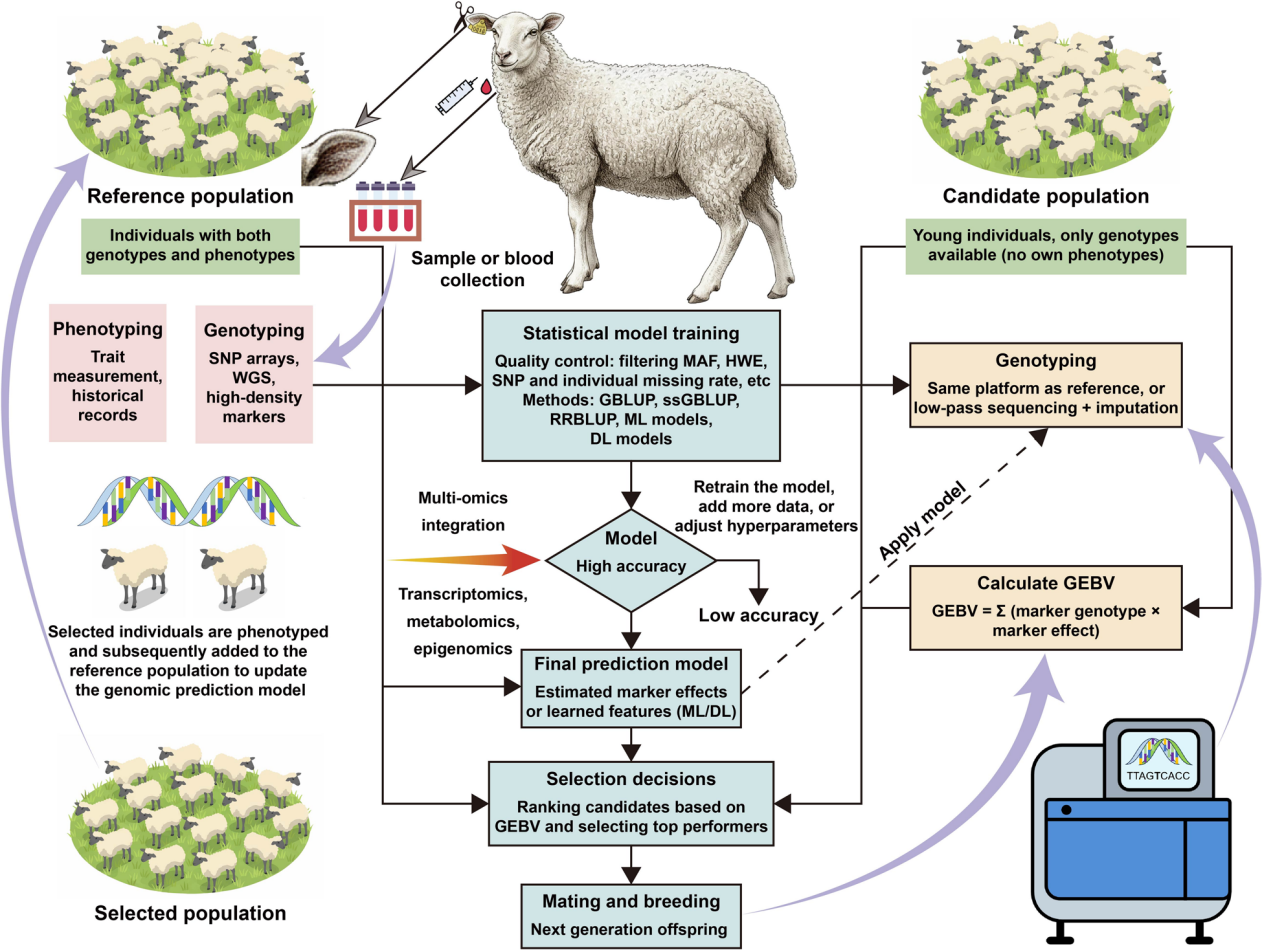
The workflow of GS in animal breeding. The reference population refers to individuals possessing both high-density genotypes and accurate phenotypes. This population serves as the training set for estimating marker effects. The candidate population consists of young individuals that are genotyped but lack phenotypic records. Their GEBVs are predicted using the trained model. The selected population comprises the top-ranking candidates derived from the candidate population based on GEBVs, which are chosen for breeding and subsequently incorporated into the reference population for model updating. Workflow: following quality control of the genotype data, the reference population is used to train statistical or ML models. These models are applied to the candidate population to calculate GEBVs. Once selected individuals have been phenotyped, the phenotypic data are integrated back into the reference population, creating a closed-loop update mechanism that improves prediction accuracy for the next generation

The fundamental objective of animal breeding is to increase the frequency of favorable alleles in populations, thereby enhancing economically important traits. Traditional methods have relied on estimating breeding values from phenotypic records and genetic relationships, primarily through pedigree-based statistical approaches. For decades, the best linear unbiased prediction (BLUP) proposed by Henderson [1], which utilizes the numerator relationship matrix, has served as the cornerstone of genetic evaluation. Advances in sequencing technologies, bioinformatics, and molecular breeding have progressively elucidated the genetic basis of complex traits, enabling the identification of underlying genes or markers. This shift facilitated the integration of marker-assisted selection (MAS), which incorporates trait-associated markers into breeding programs [2]. The advent of high-throughput genotyping technologies and the seminal proposal by Meuwissen et al. [3] in 2001 marked the formal emergence of genomic selection (GS). By replacing expected pedigree relationships with realized genomic relationships, GS directly estimates genomic estimated breeding values (GEBV), substantially reducing generation intervals and improving prediction accuracy, especially for low-heritability or hard-to-measure traits [4, 5]. A crucial factor in the success of GS is the quality and quantity of phenotypic data, particularly within the reference population used to train prediction models. Because phenotypes serve as the essential training labels that allow models to associate genotypes with trait expression, establishing a robust reference population with high-quality records is fundamental. This dependency becomes particularly critical for new traits, where accurate genomic predictions are often bottlenecked by the lack of extensive phenotypic records. Consequently, optimizing phenotyping strategies remains a pivotal challenge in maximizing GS efficiency. Another critical factor influencing the accuracy of genomic predictions is heritability, which quantifies the proportion of phenotypic variation attributable to genetic differences. Traits with higher heritability generally yield more accurate GEBVs, as the genetic signal is stronger relative to environmental noise. Conversely, low-heritability traits require larger and more precisely phenotyped reference populations to achieve comparable prediction accuracy. Understanding heritability also informs breeding program design, guiding decisions regarding reference population size and composition, phenotyping intensity, and trait selection for genomic evaluation. The workflow of GS in animal breeding is shown in Fig. 1.

As GS has become the standard in livestock breeding, its underlying statistical models have diversified to address biological complexities. The evolution began with linear mixed models, such as genomic BLUP (GBLUP) [5] and its extension, single-step GBLUP (ssGBLUP) [6, 7], which efficiently integrate genotyped and non-genotyped individuals. While robust for traits governed primarily by additive genetic effects, these models typically assume normally distributed marker effects, thereby limiting their suitability for traits influenced by major genes. Bayesian regression methods subsequently emerged to accommodate heterogeneous genetic architectures through variable shrinkage of marker effects [3, 8].

More recently, the exponential growth of phenotypic and genomic big data has pushed parametric models to their limits, driving the adoption of machine learning (ML) and deep learning (DL) in quantitative genetics [9, 10]. Unlike traditional approaches requiring explicit genetic assumptions, ML and DL learn complex patterns directly from data, offering the potential to capture non-linear interactions (e.g., dominance and epistasis), though explicitly disentangling these effects remains a challenge in DL applications. Nevertheless, these methods introduce challenges related to interpretability, overfitting, and computational demands. While DL and ML methods often demonstrate superior accuracy, their high computational complexity and extensive training times pose significant barriers to routine application. In contrast, traditional linear models like GBLUP remain the industry standard due to their computational efficiency and robustness, even if they may underutilize complex genetic architectures. Consequently, assessing the cost-effectiveness of advanced algorithms is a pivotal challenge in modern genomic evaluation. A substantial improvement in genetic gain is required to justify the increased computational resources. Beyond computational cost, adopting ML/DL approaches requires careful consideration of the trade-off between predictive performance and the risk of overfitting, as well as the need for model interpretability. Furthermore, DL models pose additional challenges related to parameter tuning and stability. Specifically, DL model hyperparameters are often unknown a priori or change dynamically during training, introducing variability in results. To achieve stable and reproducible predictions, strategies such as cross-validation, hyperparameter optimization, early stopping, dropout, and ensemble methods are commonly employed. Careful model validation is therefore essential to ensure that the added complexity of DL translates into reliable GEBVs rather than overfitting or unstable predictions.

This review systematically explores the statistical foundations of GS, tracing its evolution from classical linear and Bayesian models to emerging ML and DL techniques. By comparing their underlying assumptions, performance characteristics, and practical applicability, we aim to provide guidance for optimizing genomic evaluation in modern animal breeding.

## Linear statistical model in genomic selection

### BLUP model

The BLUP method was first proposed by Henderson [1]. However, its widespread application in breeding was delayed until the mid-1970s when advancements in computing power rendered the required complex calculations feasible. As the cornerstone of traditional genetic evaluation, BLUP operates within the framework of mixed linear models and has proven highly effective for traits with moderate to high heritability. However, for traits with low heritability, the phenotypic variance is largely dominated by environmental noise, making it difficult for BLUP to accurately separate the genetic signal from the residual effects based solely on phenotypic records. Furthermore, for traits that are difficult or expensive to measure, the scarcity of phenotypic data limits the model's statistical power, leading to reduced selection efficiency and accuracy [11]. The fundamental mixed linear model is expressed as:

1 $$\begin{array}{c}\boldsymbol{y}=\boldsymbol{Xb}+\boldsymbol{Za}+\boldsymbol{e},\end{array}$$

where $\boldsymbol{y}$ is the vector of observations; $\boldsymbol{b}$ and $\boldsymbol{a}$ represent fixed and random effects, respectively; $\boldsymbol{X}$ and $\boldsymbol{Z}$ are their incidence matrices; and $\boldsymbol{e}$ is the vector of random residuals. To estimate these effects, Henderson [1] derived the mixed model equations (MME):

2 $$\begin{array}{c}\left[\begin{array}{cc}\boldsymbol{X}{\prime}\boldsymbol{R}^{-1}\boldsymbol{X}& \boldsymbol{X}{\prime}\boldsymbol{R}^{-1}\boldsymbol{Z}\\ \boldsymbol{Z}{\prime}\boldsymbol{R}^{-1}\boldsymbol{X}& \boldsymbol{Z}{\prime}\boldsymbol{R}^{-1}\boldsymbol{Z}+\boldsymbol{G}^{-1}\end{array}\right]\left[\begin{array}{c}\widehat{\boldsymbol{b}}\\ \widehat{\boldsymbol{a}}\end{array}\right]=\left[\begin{array}{c}\boldsymbol{X}{\prime}\boldsymbol{R}^{-1}y\\ \boldsymbol{Z}{\prime}\boldsymbol{R}^{-1}\boldsymbol{y}\end{array}\right],\end{array}$$

when assuming $\boldsymbol{R}=\boldsymbol{I}{\sigma}_{e}^{2}$ and $\boldsymbol{G}=\boldsymbol{A}{\sigma}_{a}^{2}$ (where $\boldsymbol{A}$ is the additive genetic relationship matrix), the MME can be simplified to:

3 $$\begin{array}{c}\left[\begin{array}{cc}\boldsymbol{X}{\prime}\boldsymbol{X}& \boldsymbol{X}{\prime}\boldsymbol{Z}\\ \boldsymbol{Z}{\prime}\boldsymbol{X}& \boldsymbol{Z}{\prime}\boldsymbol{Z}+\boldsymbol{A}^{-1}k\end{array}\right]\left[\begin{array}{c}\widehat{\boldsymbol{b}}\\ \widehat{\boldsymbol{a}}\end{array}\right]=\left[\begin{array}{c}\boldsymbol{X}{\prime}\boldsymbol{y}\\ \boldsymbol{Z}{\prime}\boldsymbol{y}\end{array}\right], k=\frac{{\sigma}_{e}^{2}}{{\sigma}_{a}^{2}}.\end{array}$$

### GBLUP model

VanRaden [5] proposed the GBLUP method, offering a robust extension of the traditional BLUP framework. This approach utilizes a mixed linear model in which the pedigree relationship matrix is replaced by a genomic relationship matrix ($\boldsymbol{G}$) constructed from high-density single nucleotide polymorphism (SNP) markers. The GBLUP linear model is expressed as:

4 $$\begin{array}{c}\boldsymbol{y}=\boldsymbol{Xg}+\boldsymbol{Za}+\boldsymbol{e},\end{array}$$

where $\boldsymbol{y}$ denotes the vector of observed values; $\boldsymbol{X}$ represents the matrix of observed values for the genotype ($\boldsymbol{g}$) at a single locus; $\boldsymbol{Z}$ is the association matrix for the additive effect of genes, where $\boldsymbol{a}$ denotes the additive effect of genes; $\boldsymbol{e}$ denotes the residual vector. The corresponding MME is as follows:

5 $$\begin{array}{c}\left[\begin{array}{cc}\boldsymbol{X}{\prime}\boldsymbol{X}& \boldsymbol{X}{\prime}\boldsymbol{Z}\\ \boldsymbol{Z}{\prime}\boldsymbol{X}& \boldsymbol{Z}{\prime}\boldsymbol{Z}+\boldsymbol{G}^{-1}k\end{array}\right]\left[\begin{array}{c}\widehat{\boldsymbol{g}}\\ \widehat{\boldsymbol{a}}\end{array}\right]=\left[\begin{array}{c}\boldsymbol{X}{\prime}\boldsymbol{y}\\ \boldsymbol{Z}{\prime}\boldsymbol{y}\end{array}\right], k=\frac{{\sigma}_{e}^{2}}{{\sigma}_{a}^{2}}.\end{array}$$

The $\boldsymbol{G}$ matrix is constructed from all SNP markers. Assuming $\boldsymbol{a}=\boldsymbol{Zg}$, then $Var(\boldsymbol{a})=Z{Z}{\prime}{\sigma}_{g}^{2}$, ${\sigma}_{a}^{2}=\frac{{\sigma}_{g}^{2}}{2\sum {p}_{i}(1-{p}_{i})}$. The $\boldsymbol{Z}\boldsymbol{Z}{\prime}$ matrix can be normalized to yield $\boldsymbol{G}=\frac{\boldsymbol{Z}{\boldsymbol{Z}}{\prime}}{2\boldsymbol{\sum} {p}_{i}(1-{p}_{i})}$ and $Var(\boldsymbol{a})=\boldsymbol{G}{\sigma}_{a}^{2}$, where ${p}_{i}$ denotes the minimum allele frequency at a locus i, and $\boldsymbol{Z}$ represents the individual genotype matrix. In addition to the method proposed by VanRaden [5], researchers have explored other approaches for constructing matrices. For instance, Goddard et al. [12] proposed a blended $\boldsymbol{G}$ matrix, $\boldsymbol{G}=\boldsymbol{A}+b(\boldsymbol{G}_{m}-\boldsymbol{A})$, to incorporate pedigree information, thereby enhancing the flexibility of GBLUP for varied genetic architectures. Yang et al. [13] proposed calculating the $\boldsymbol{G}$ matrix based on weights:

6 $$\begin{array}{c}\boldsymbol{A}_{jk}=\frac{1}{N}{\sum}_{i}\boldsymbol{A}_{ijk}=\left\{\begin{array}{c}\frac{1}{N}{\sum}_{i}\frac{({x}_{ij}-2{p}_{i})({x}_{ik}-2{p}_{i})}{2p(1-{p}_{i})}, j\ne k\\ 1+\frac{1}{N}{\sum}_{i}\frac{{x}_{ij}^{2}-(1+2{p}_{i}){x}_{ij}+2{p}_{i}^{2}}{2{p}_{i}(1-{p}_{i})}, j=k\end{array}\right..\end{array}$$

These approaches differ primarily in their assumptions regarding the distribution of marker effects. The standard method proposed by VanRaden [5] assumes that all markers contribute equally to the genetic variance, which is computationally efficient and robust for highly polygenic traits. However, it may yield suboptimal results for traits influenced by major genes. To address data limitations, Goddard et al. [12] introduced a blended matrix incorporating pedigree information, which enhances the stability of variance component estimation, particularly in populations with limited sample sizes or weak genetic connections. In contrast, the weighting strategy suggested by Yang et al. [13] allows for heterogeneous variances across markers. This approach is advantageous for livestock populations where complex traits are often governed by a few loci with large effects, as it assigns higher weights to influential SNPs, thereby improving prediction accuracy compared to the equal-weight assumption. In GS, multi-trait GBLUP models often provide more accurate GEBVs than single-trait models by leveraging genetic correlations [14]. Wang et al. [15] further noted that modifying kinship derivation enables GBLUP to maintain significant computational advantages, especially for complex traits. However, a fundamental limitation of standard GBLUP is its assumption that all markers contribute equally to genetic variance. In reality, genomic landscapes are often dominated by a few large-effect loci, while most markers have minor effects. This discrepancy was addressed by Zhang et al. [16] through the trait-specific marker derived relationship matrix BLUP (TABLUP), which integrates marker effect estimation with matrix construction by assigning trait-specific weights to individual SNPs. Haque et al. [17] demonstrated the substantial potential of iterative weighted GBLUP in predicting complex carcass traits within a cohort of nearly 20,000 Hanwoo cattle. Using real phenotypic and genotypic data, this study highlights the practical applicability of the method in livestock breeding programs. By iteratively reweighting SNP effects to enhance the contribution of influential loci, the weighted GBLUP model achieved a predictive accuracy improvement of up to 8.97% relative to conventional GBLUP. This performance closely approached that of more computationally demanding Bayesian approaches, offering a favorable balance between prediction accuracy and computational efficiency. However, a limitation of this iterative approach is its dependence on the quality of initial variance estimates, and it may require careful monitoring to ensure convergence.

### ssGBLUP model

**Fig. 2:**
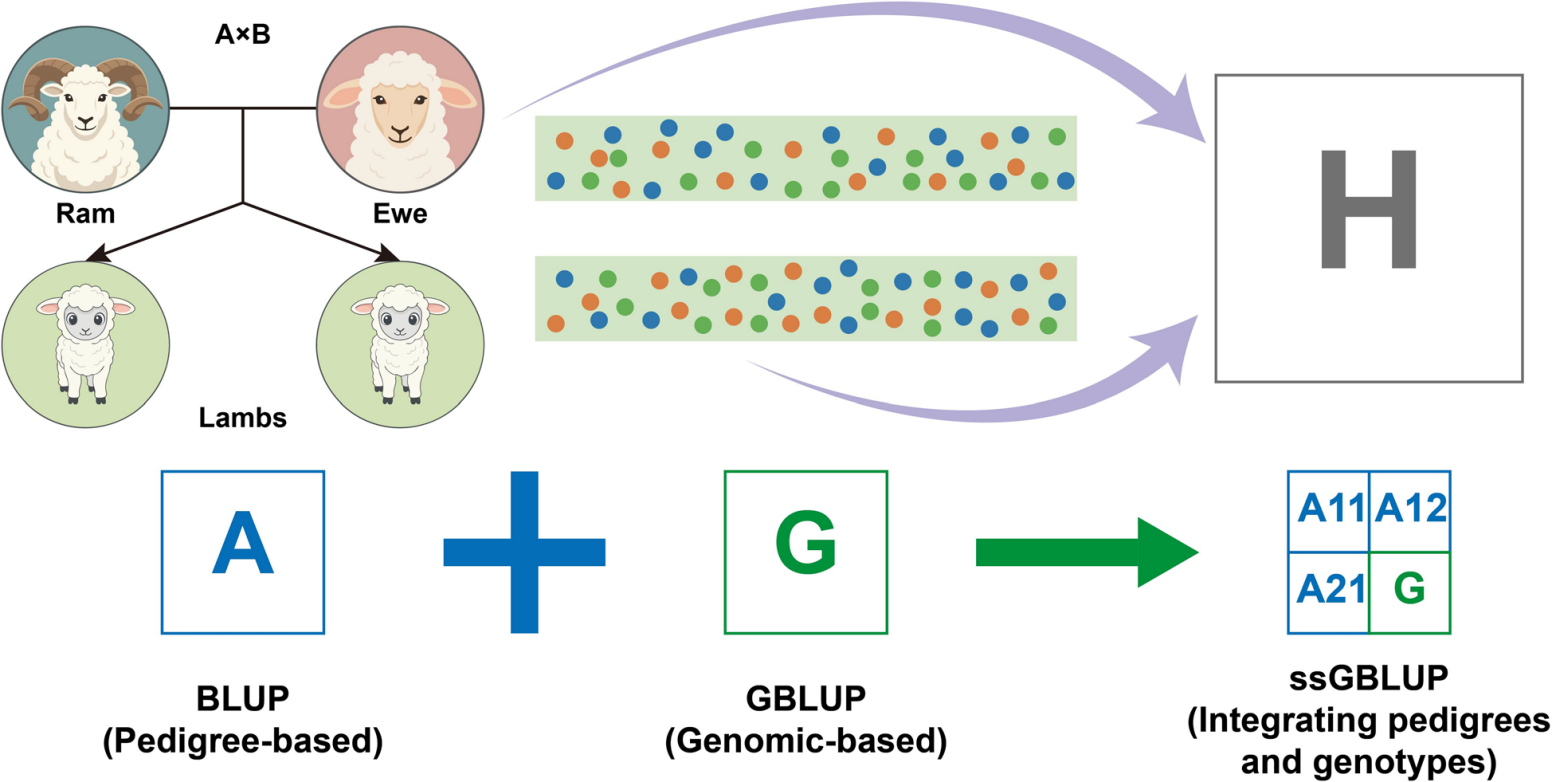
Schematic of relationship matrix construction in linear mixed models. The diagram illustrates the integration of independently derived relationship matrices. It depicts the transition from the traditional pedigree-based BLUP ($\boldsymbol{A}$ matrix) and genomic-based GBLUP ($\boldsymbol{G}$ matrix) to the integrated ssGBLUP. By constructing the $\boldsymbol{H}$ matrix, which combines both pedigree and genomic information, the model enables a more comprehensive and accurate estimation of breeding values for both genotyped and non-genotyped individuals

In livestock breeding practice, constraints such as high genotyping costs often result in a significant number of individuals having pedigree and phenotypic records but lacking genotype data. To utilize this diverse information, Misztal et al. [18] proposed a theoretical framework to integrate pedigree, genotype, and phenotype data. Subsequently, Legarra et al. [6] and Christensen et al. [7] independently derived methods to construct a unified kinship matrix ($\boldsymbol{H}$) by merging the pedigree relationship matrix ($\boldsymbol{A}$) and the genomic relationship matrix ($\boldsymbol{G}$). This integration is particularly effective in livestock populations, which are typically characterized by extensive linkage disequilibrium (LD) blocks due to historical bottlenecks and selection. Such an extensive LD structure allows markers to effectively capture the effects of quantitative trait loci (QTLs) even at a distance, ensuring that the measurable genetic effects are well-distributed and captured across the genome within the $\boldsymbol{H}$ matrix framework. This ssGBLUP method enables the simultaneous estimation of breeding values for both genotyped and non-genotyped individuals, significantly expanding the applicability of GBLUP methods [19]. The structural evolution from the $\boldsymbol{A}$ and $\boldsymbol{G}$ matrices to the combined $\boldsymbol{H}$ matrix is systematically illustrated in Fig. 2. The $\boldsymbol{H}$ matrix is constructed as follows [18]:

7 $$\begin{array}{c}\boldsymbol{H}=\left[\begin{array}{cc}\boldsymbol{A}_{11}\boldsymbol-{A}_{12}\boldsymbol{A}_{22}^{-1}\boldsymbol{A}_{21}+\boldsymbol{A}_{12}\boldsymbol{A}_{22}G\boldsymbol{A}_{22}^{-1}\boldsymbol{A}_{21}& \boldsymbol{A}_{12}\boldsymbol{A}_{22}^{-1}\boldsymbol{G}\\ \boldsymbol{G}\boldsymbol{A}_{22}^{-1}\boldsymbol{A}_{21}& \boldsymbol{G}\end{array}\right],\end{array}$$

where $\boldsymbol{A}_{11}$ and $\boldsymbol{A}_{22}$ represent the pedigree sub-matrices for non-genotyped and genotyped individuals, respectively. To address potential singularity issues that would otherwise make the $\boldsymbol{G}$ matrix non-invertible, a blending approach is typically employed: $\boldsymbol{G}_{w}=(1-w)\boldsymbol{G}+w\boldsymbol{A}_{22}$, where w is the weighting factor representing the proportion of polygenic inheritance effects. Given the computational burden of directly inverting the $\boldsymbol{H}$ matrix, Christensen et al. [7] derived a simplified form for its inverse: $$\boldsymbol{H}^{-1}=\boldsymbol{A}^{-1}+\left[\begin{array}{cc}0& 0\\ 0& \boldsymbol{G}^{-1}\boldsymbol{-{A}}_{22}^{-1}\end{array}\right]$$. Following the construction of $\boldsymbol{H}^{-1}$, the process of solving the MME remains consistent with traditional BLUP:

8 $$\begin{array}{c}\left[\begin{array}{cc}\boldsymbol{X}{\prime}\boldsymbol{X}& \boldsymbol{X}{\prime}\boldsymbol{Z}\\ \boldsymbol{Z}{\prime}\boldsymbol{X}& \boldsymbol{Z}{\prime}\boldsymbol{Z}+\boldsymbol{H}^{-1}k\end{array}\right]\left[\begin{array}{c}\widehat{\boldsymbol{b}}\\ \widehat{\boldsymbol{a}}\end{array}\right]=\left[\begin{array}{c}\boldsymbol{X}{\prime}y\\ \boldsymbol{Z}{\prime}\boldsymbol{y}\end{array}\right], k=\frac{{\sigma}_{e}^{2}}{{\sigma}_{a}^{2}}.\end{array}$$

Despite its widespread adoption, ssGBLUP faces challenges regarding the compatibility between the $\boldsymbol{G}$ and $\boldsymbol{A}$ matrices. This incompatibility stems primarily from incomplete pedigree records and discrepancies in allele frequency definitions [20]. Specifically, ssGBLUP assumes that the allele frequencies used to construct the $\boldsymbol{G}$ matrix represent the unselected base population, which is often difficult to define in practical settings. Failure to properly account for the stratification of allele frequencies across different generations or sub-populations results in a fundamental incompatibility between the $\boldsymbol{G}$ and $\boldsymbol{A}$ matrices. This discrepancy introduces substantial bias into the MME, typically manifesting as the inflation or deflation of GEBVs and a biased estimation of genetic variance, thereby compromising the prediction accuracy for both genotyped and non-genotyped individuals. To mitigate this, Christensen [21] introduced parameters γ and s to correct for population bias. Furthermore, to address the lack of pedigree connections in multi-population evaluations, Legarra et al. [22] proposed the metafounders theory. Studies on cross-population genomic prediction have demonstrated that the metafounder approach effectively captures ancestral correlations, yielding predictive accuracies at least as high as standard ssGBLUP models that assume unrelated populations [23, 24]. Beyond the frequentist framework, Fernando et al. [25] introduced single-step Bayesian regression (ssBR). Recent evaluations in buffalo populations suggest that ssBR variants can outperform ssGBLUP in predicting polygenic traits, especially when genotyping resources are limited [26].

To further overcome the limitations of standard ssGBLUP, which assumes equal variance for all markers and may fail to capture major genes embedded in a polygenic background, recent research has shifted toward integrating genome-wide association study (GWAS) results into the single-step framework. Pang et al. [27] introduced the single-step genome-wide association assisted BLUP model, which relaxes the infinitesimal assumption by assigning differential weights to SNPs based on GWAS-derived effect sizes. Their empirical findings, derived from both simulation scenarios and real pig data, demonstrated that the single-step genome-wide association assisted BLUP with pseudo-quantitative trait nucleotides as covariates significantly outperforms both standard ssGBLUP and iterative weighted ssGBLUP. These markers are identified through GWAS and LD pruning. Crucially, this method enhances prediction accuracy not only for traits with large-effect loci but also remains effective for complex, polygenic traits by capturing a broader spectrum of genetic variance through weighted markers. Despite the development of these advanced models, standard ssGBLUP continues to demonstrate robust practical utility. For instance, Mancisidor et al. [28] observed that ssGBLUP improved the accuracy of breeding value predictions for fiber diameter standard deviation in Huacaya alpacas by up to 6.44% compared to traditional BLUP, even with limited genotypic records. Addressing the persistent challenges of pedigree incompleteness and base population definition, Himmelbauer et al. [29] used a simulated cattle population to theoretically demonstrate that the metafounder approach significantly reduces evaluation bias and dispersion compared to traditional unknown-parent groups in scenarios with fragmented pedigrees. By accurately aligning pedigree and genomic relationships, the metafounder framework, enhanced by genotype-based grouping, offers a superior strategy for managing complex population structures in modern genomic evaluations.

### RRBLUP model

Traditional least squares methods typically treat marker effects as fixed. However, in genomic prediction, the number of SNP markers (p) often far exceeds the sample size (n), leading to multicollinearity and making the simultaneous estimation of all marker effects via ordinary least squares mathematically intractable. To circumvent this, early approaches relied on selecting only a small subset of significant SNPs, which often led to overfitting and failed to capture the small effects of rare variants. To address these limitations, Whittaker et al. [30] proposed a ridge regression approach, now commonly known as ridge regression BLUP (RRBLUP). Unlike least squares, RRBLUP treats all SNP effects as random variables following a normal distribution with a constant variance. By employing a linear mixed model, RRBLUP estimates the effect of every marker simultaneously and calculates the GEBVs as the sum of these effects. In GS, RRBLUP is categorized as an indirect approach (estimating marker effects first), while GBLUP is a direct approach (estimating individual relationships directly). Although the two methods are mathematically equivalent and yield comparable accuracy under Gaussian assumptions, their computational efficiency differs based on data dimensions. Since p≫n in most modern genomic datasets [31], RRBLUP is generally more computationally demanding and time-consuming than GBLUP.

Recent empirical studies have continued to highlight the utility and limitations of RRBLUP in animal breeding programs. A study on Rhode Island Red chickens genotyped with a 50 K SNP array integrated ML approaches with classical methods and compared their performance with RRBLUP and BayesA across 10 economic traits. While ML models generally outperformed RRBLUP for traits such as body weight and eggshell strength, RRBLUP and BayesA exhibited 2%–58% higher predictive accuracy for egg number traits, highlighting the robustness of RRBLUP for traits with a predominantly additive genetic architecture. Notably, the incorporation of GWAS significant SNPs marked a ‘sweet spot' where the advantage of RRBLUP was most pronounced (up to 58%), whereas standard predictions showed more marginal differences [32]. Similarly, Sahebalam and Gholizadeh [33] evaluated different approaches for estimating the shrinkage parameter in RRBLUP using pig genomic datasets, finding that the allelic frequencies RRBLUP variant delivered comparable or superior predictive accuracy while substantially reducing computational burden. However, such accuracy gains can sometimes be attributed to overfitting to the specific dataset rather than representing a general methodological advance, underscoring the importance of validation in independent populations.

### Bayesian model

Bayesian methods typically assume a sparse genetic architecture where only a small proportion of SNPs are linked to QTLs with large effects, while the majority have negligible or no effect. The linear model is defined as:

9 $$\boldsymbol{y}=\boldsymbol{Xb}+{\sum}_{j=1}^{M}\boldsymbol{Z}_{j}{\alpha}_{j}+\boldsymbol{e},$$

where $\boldsymbol{y}$ is the vector of phenotypic observations, $\boldsymbol{b}$ is vector of fixed effects, M is the number of markers, ${\alpha}_{j}$ is the effect of the j-th marker, $\boldsymbol{X}$ and $\boldsymbol{Z}_{j}$ are the incidence matrices of $\boldsymbol{b}$ and ${\alpha}_{j}$, respectively, and $\boldsymbol{e}$ is the vector of random residuals. The residuals follow a normal distribution $N(0, \boldsymbol{I}{\sigma}_{e}^{2})$, where $\boldsymbol{I}$ is the identity matrix and ${\sigma}_{e}^{2}$ is the residual variance. Solving the model yields an estimate of the marker effect α, subsequently, the GEBV for individual i is calculated as $gEB{V}_{i}=\sum {Z}_{ij}{\alpha}_{j}$, where ${Z}_{ij}$ is the genotype of individual i at locus j, and ${\alpha}_{j}$ is the estimated effect of locus j.

To enhance estimation efficiency, Mathew et al. [34] applied Markov chain Monte Carlo (MCMC) theory, followed by the introduction of Gibbs sampling to address computational complexity. These developments led to the "Bayesian Alphabet" family of models, widely used for GEBV prediction, including BayesA [3], BayesB [3], BayesC [8], BayesC*π* [8], BayesD*π* [8], and Bayesian Lasso [35]. To better capture complex genetic architectures and incorporate prior biological information, the "Bayesian Alphabet" has continuously evolved, with many models derived directly from one another. Further advancements led to BayesR [36], which utilizes a mixture of four normal distributions to categorize SNP effects into null, small, medium, and large classes, offering enhanced predictive accuracy for traits with varying genetic architectures. Building upon BayesR, the BayesRC [37] model incorporates prior biological knowledge by classifying markers into distinct functional categories. Most recently, models such as BayesRCO [38] have extended this framework to handle overlapping functional annotations, allowing multi-annotated SNPs to be modeled more accurately [39]. This evolution highlights a clear transition from uniform genomic priors to highly flexible, biologically informed architectures.

### BayesA and BayesB models

BayesA, proposed by Meuwissen et al. [3], utilizes a multi-level prior distribution where SNP effects ${g}_{i}$ follow a normal distribution $N(0, {\sigma}_{gi}^{2})$, and the variance ${\sigma}_{gi}^{2}$ follows an inverse Chi-square distribution ${\chi }^{-2}(\nu , S)$. Meuwissen et al. [3] set the degrees of freedom ν and scale parameter S at 4.012 and 0.002, respectively, while others have explored alternative priors [40, 41]. The resulting posterior distribution of the effect variance ${\sigma}_{gi}^{2}$ is also an inverse Chi-square distribution ${\chi }^{-2}(\nu +{n}_{i}, {g}_{i}{\prime}{g}{\prime})$ [42].

BayesB was developed to better reflect genomic reality by assuming most segments have no effect. It introduces a parameter *π* representing the probability that a marker has zero effect and uses a mixture distribution for marker variances. While BayesA assumes all SNPs have effects (*π* = 0), BayesB is more accurate for traits controlled by a few large-effect QTLs [3, 43]. Due to its complex posterior, BayesB requires a combination of Gibbs and Metropolis–Hastings (MH) sampling [3]. The prior distribution assumptions in BayesB are as follows:

10 $$\begin{array}{c}{\sigma}_{{g}_{i}}^{2}=0\;with\;probability\;\pi ,\end{array}$$

11 $$\begin{array}{c}\sigma_{g_i}^2\sim\chi^{-2}(\nu,S)\:with\:probability\:(1-\pi).\end{array}$$

Habier et al. [8] proposed deriving S when ν=4.2. Since $E\left({\sigma}_{{a}_{k}}^{2}\right)=\frac{{\nu}_{a}{s}_{a}^{2}}{{\nu}_{a}-2}={\widetilde{\sigma }}_{a}^{2}$, it follows that ${S}_{a}^{2}=\frac{{\widetilde{\sigma }}_{a}^{2}\left({\nu}_{a}-2\right)}{{\nu}_{a}}$. Here, ${\widetilde{\sigma }}_{a}^{2}$ is the variance of the additive effect for a randomly sampled locus, which can be related to the additive-genetic variance explained by the SNP, ${\widetilde{\sigma }}_{s}^{2}$ as:

12 $$\begin{array}{c}{\widetilde{\sigma }}_{a}^{2}=\frac{{\widetilde{\sigma }}_{s}^{2}}{\left(1-\pi \right)\sum_{k=1}^{K}2{p}_{k}\left(1-{p}_{k}\right)},\end{array}$$

where ${p}_{k}$ denotes the allele frequency of SNP k, while the three parameters ν, S, and *π* are 4.2339, 0.0429, and 0.947, respectively [3]. The choice of these prior parameters directly impacts the model's output. By changing the priors from fixed, arbitrary values to empirical values derived from actual genetic variance, several key differences can be expected in the results. First, it alters the estimated variance of individual markers, allowing the model to more accurately distinguish between loci with major effects and background polygenic noise. Second, it allows the model to adapt flexibly to different genetic architectures across various traits. Ultimately, these differences lead to improved prediction accuracy for the GEBVs.

### BayesC, BayesCπ, and BayesDπ models

To reduce the subjectivity associated with manually setting *π*, Habier et al. [8] proposed several optimized methods, including BayesC, BayesC*π*, and BayesD*π*. The BayesC method utilizes a fixed *π* and assumes a common variance for all SNPs that possess an effect. In contrast, BayesC*π* treats *π* as an unknown parameter following a uniform prior distribution. Further extending this flexibility, BayesD*π* estimates both *π* and the scale parameter S from the data. Notably, the performance of BayesC*π* is highly sensitive to factors such as heritability and marker density, which makes it particularly suitable for analyzing traits with medium-to-high heritability [44].

### Bayesian Lasso model

The Bayesian Lasso (BL), proposed by Park and Casella [35], obtains linear regression coefficients by normalizing predictor variables ${x}_{ij}$ and centering the response values ${y}_{i}$. It addresses the ${l}_{1}$ penalty regression problem by minimizing the following objective function with respect to $\boldsymbol{\beta} =\{{\beta}_{j}\}$:

13 $$\begin{array}{c}\sum_{i=1}^{N}{\left({y}_{i}-{\sum}_{j}{x}_{ij}{\beta}_{j}\right)}^{2}+\lambda \sum_{j=1}^{p}\left|{\beta}_{j}\right|.\end{array}$$

This is equivalent to a constrained sum of squares minimization: $\sum |\beta_{j}|\le s$. A distinguishing feature of BL is the assumption that marker effect variances follow an exponential distribution, resulting in a Laplace prior for the marker effects. This Laplace distribution is particularly effective for genomic prediction as it assigns higher probabilities to extreme values, whether large or small [45]. To systematically compare the differences among these methods, their prior distribution assumptions regarding the key parameters *π*, SNP effects, and their variances are summarized in Table 1.

**Table 1:** Comparison of prior distribution assumptions in different Bayesian models

Bayesian models	Prior distribution of *π*	Prior distribution of${g}_{i}$	Prior distribution of${\sigma}_{gi}^{2}$
BayesA	0	${g}_{i}\sim N(0, {\sigma}_{gi}^{2})$	${\sigma}_{gi}^{2}\sim {\chi }^{-2}(\nu , S)$
BayesB	Fixed value	${g}_{i}\sim N(0, {\sigma}_{gi}^{2})$	${\sigma}_{gi}^{2}\sim {\chi }^{-2}(\nu , S)$
BayesC	Fixed value	${g}_{i}\sim N(0,{ \sigma }_{g}^{2})$	${\sigma}_{g}^{2}\sim {\chi }^{-2}(\nu , S)$
BayesC*π*	U(0, 1)	${g}_{i}\sim N(0, {\sigma}_{g}^{2})$	${\sigma}_{g}^{2}\sim {\chi }^{-2}(\nu , S)$
BayesD*π*	U(0, 1)	${g}_{i}\sim N(0, {\sigma}_{g}^{2})$	${\sigma}_{g}^{2}\sim {\chi }^{-2}(\nu , Gamma(1, 1))$
Bayesian LASSO	0	${g}_{i}\sim N(0,{ \sigma }_{gi}^{2})$	${\sigma}_{gi}^{2}\sim Exp({\lambda }^{2}/2)$

The primary challenge in applying these Bayesian methods lies in the formulation of appropriate prior distributions for hyperparameters. While Bayesian models typically offer improved prediction accuracy over BLUP-based methods by estimating a larger set of parameters, they impose a significantly higher computational burden [46]. The MCMC process is particularly intensive, requiring tens of thousands of iterations where all marker effects must be re-estimated in each cycle. This sequential nature consumes substantial time, often conflicting with the stringent timeliness requirements of practical animal and plant breeding [47].

To enhance computational efficiency, various optimized versions have been developed, such as fastBayesA [48], fBayesB [49], wBSR [50], emBayesR [51], EBL [52], BayesRS [53], and BayesTA [54]. Despite these advancements, the classical "Bayesian Alphabet" remains the most prevalent in current GS practices. Ultimately, the predictive success of these models depends on the degree to which their underlying prior assumptions align with the actual genetic architecture of the target trait [55]. Recent empirical studies in diverse livestock populations have further substantiated these theoretical expectations. In a comprehensive 2025 study of over 16,000 Chinese Holstein cattle, BayesR emerged as the most robust model for production traits, achieving a peak predictive accuracy of 0.625—outperforming both GBLUP and several SNP-weighted models [56]. This superiority is particularly pronounced for traits with known major-effect QTLs, such as milk fat and protein yield, where Bayesian models more effectively capture the non-infinitesimal genetic architecture. Furthermore, in the genomic evaluation of Alpine Merino sheep, research revealed that while GBLUP is sufficient for low-heritability traits, BayesA and BayesB provide a significant accuracy advantage as trait heritability and marker density increase, particularly when utilizing high-density SNP arrays [57]. Additionally, for complex reproductive traits in Hanwoo cattle, recent applications of BL and BayesR recorded a 2% to 6% improvement in prediction reliability over traditional linear methods [58]. These findings collectively indicate that the Bayesian framework remains a cornerstone of GS, offering an optimal balance of biological interpretability and predictive power across different species and genetic architectures. However, as genomic datasets grow exponentially in size and increasingly incorporate highly non-linear multi-omics layers, traditional parametric models face significant computational and structural bottlenecks. This escalating data complexity has catalyzed the exploration of non-parametric ML approaches, which offer novel solutions to these emerging challenges.

## Machine learning approaches in genomic selection

**Fig. 3:**
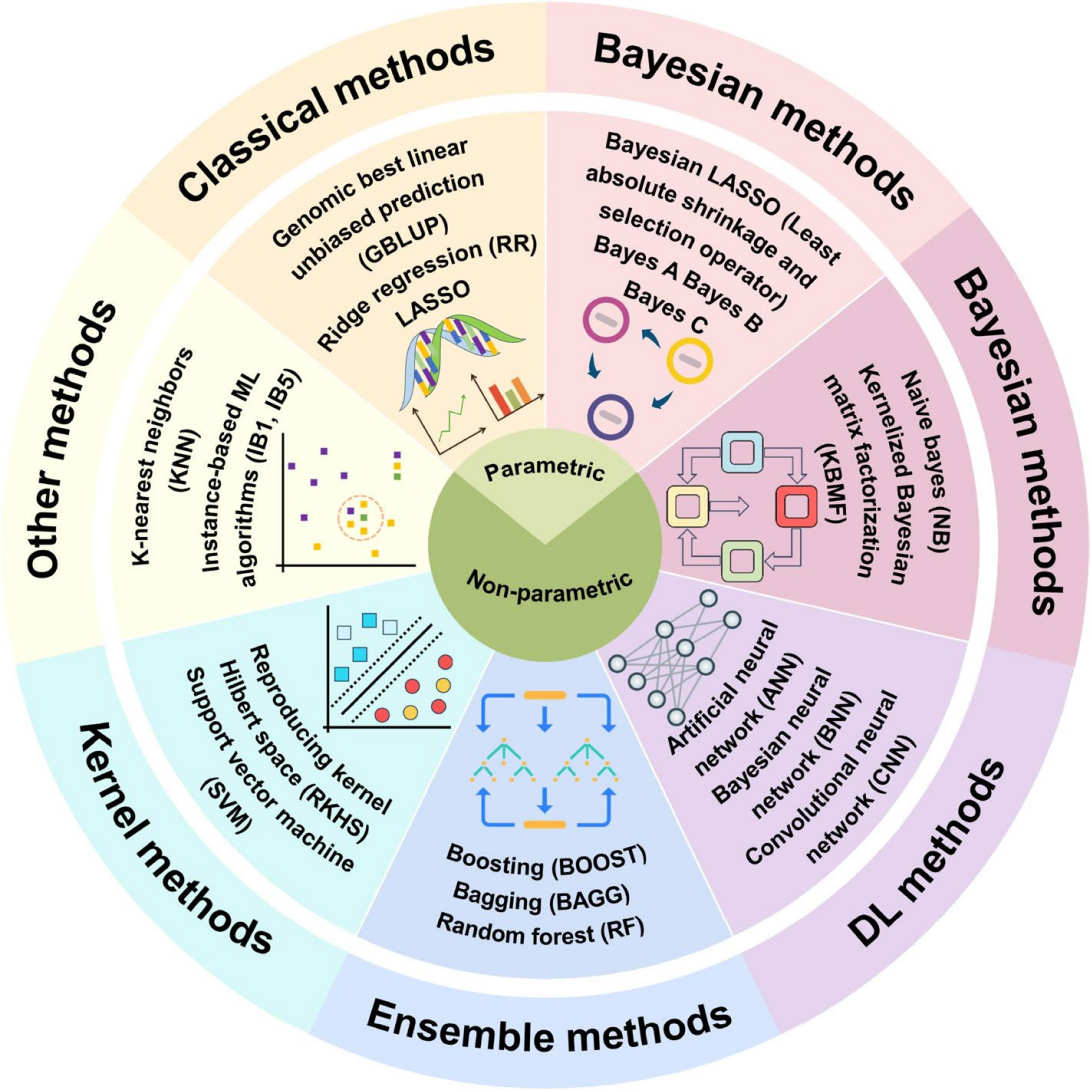
Classification of the most popular parametric and non-parametric ML regression models. This diagram provides a comprehensive taxonomy of genomic prediction models utilized in animal biotechnology. At the center, models are bifurcated into parametric and non-parametric approaches. The upper hemisphere details established statistical frameworks, including classical methods and Bayesian methods. The lower hemisphere illustrates the expanding landscape of ML, featuring kernel methods, ensemble methods, and DL architectures. This categorization serves as a roadmap for selecting optimal computational tools to enhance the accuracy of GEBV across diverse livestock populations

Besides parametric models based on BLUP and Bayesian theory, GS increasingly incorporates non-parametric ML approaches. Unlike traditional parametric and Bayesian linear regression, ML models do not necessitate explicit assumptions regarding the underlying data distribution. These models learn complex patterns directly from training populations to predict phenotypes in target populations with similar genetic architectures [59]. ML methods are primarily classified into supervised and unsupervised learning [60], with mainstream prediction techniques in GS encompassing neural networks, ensemble methods, and kernel-based approaches [61]. As summarized in Fig. 3, these non-parametric models offer distinct advantages in handling high-dimensional and non-linear biological data. However, their application is not without challenges. ML models may struggle with the adequate representation of rare alleles due to data sparsity, and they carry an inherent risk of overfitting during the learning process if regularization is not strictly applied. Furthermore, the "black-box" nature of some ML algorithms can obscure the biological interpretability of the genotype-to-phenotype prediction mechanisms. These aspects will be explored in detail in this chapter.

### Decision tree

The decision tree (DT) is a widely used supervised learning algorithm primarily employed for classification and regression tasks based on recursive partitioning [62]. First introduced by Breiman et al. [63], this method partitions large heterogeneous datasets into multiple smaller homogeneous subsets to form a hierarchical structure. In this tree structure, each intermediate node corresponds to a feature, each branch represents a value of that feature, and each terminal node stores a specific category or a regression function [64]. Following the development of the ID3 and C4.5 algorithms by Quinlan [65], the classification and regression tree (CART) method emerged as a significant advancement, utilizing the Gini impurity to identify optimal segmentation points [64, 66]. A recent study by Liu et al. [67] in yellow-feathered broilers revealed that tree-based ensemble methods markedly outperformed traditional GBLUP and Bayesian approaches, achieving over 60% improvement in predictive accuracy for carcass traits. This suggests that while individual trees provide a robust foundation for modeling nonlinear relationships, they are often prone to high variance and overfitting. Therefore, their integration into ensemble systems is essential. By aggregating predictions from multiple diverse trees, ensemble methods significantly reduce model variance and improve generalization ability, thereby optimizing performance in complex GS scenarios characterized by intricate genetic architectures.

### Ensemble learning

Ensemble learning is a supervised learning approach that constructs and combines multiple weak learners to form a comprehensive strong learner, also known as a multi-classifier system. Common strategies include bagging, boosting, and stacking [68–70]. In GS, ensemble methods often outperform single ML models by mitigating individual model biases. However, they may require more extensive tuning of structural and training hyperparameters (e.g., learning rate, number of estimators, tree depth) to optimize model architecture and prevent overfitting, which differs from the hyperparameter prior specification in Bayesian frameworks [71].

### Bagging

**Fig. 4:**
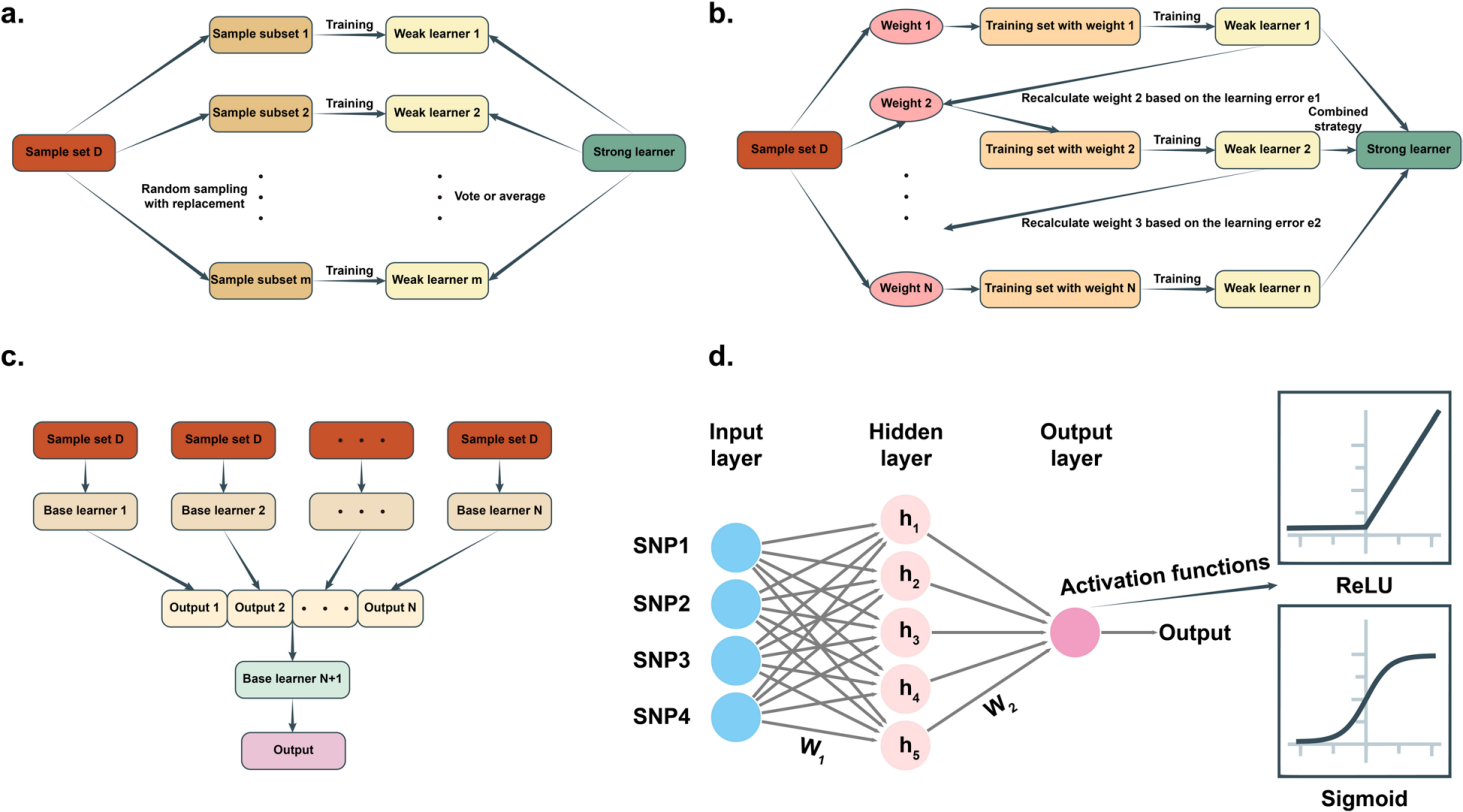
Schematic architectures of ensemble learning and neural networks frameworks in genomic prediction. This diagram illustrates the implementation of advanced computational frameworks for GS. **a** Bagging utilizes parallel weak learners to ensure model robustness through random sampling. **b** Boosting optimizes prediction accuracy via sequential weight adjustment based on learning errors. **c** Stacking achieves superior performance by consolidating outputs from multiple base models into a meta-learner. **d** MLP processes high-dimensional SNP data through hierarchical layers and nonlinear activation functions (sigmoid and ReLU) to capture complex genetic effects

Bagging involves training *m* base learners independently on data subsets generated via random sampling with replacement from the original dataset *D*, a process known as bootstrap sampling that resamples the observations (rows) of the dataset [68]. The final prediction is obtained through voting or averaging (Fig. 4a). This parallelized approach significantly reduces prediction variance and is robust against data noise [72]. However, it is important to note that when dealing with unbalanced datasets, standard bootstrap sampling can inadvertently generate subsets with skewed class distributions (e.g., lacking minority class samples), potentially introducing bias into the model. Therefore, specialized sampling strategies or weighting schemes are often required in such scenarios to ensure robust performance. Nevertheless, bagging remains sensitive to the randomness inherent in bootstrap sampling, and its overall stability can still be influenced by fluctuations in the training data [73].

### Boosting

Boosting iteratively trains a series of weak learners, where each subsequent model focuses on the samples misclassified or poorly predicted by its predecessor by adjusting instance weights [69, 74] (Fig. 4b). One classic implementation is adaptive boosting (Adaboost) [75], which assigns higher weights to hard-to-classify samples and determines the final output through a weighted decision process. Adaboost.RT is an enhanced version specifically optimized for regression tasks in biological data [76]. Recent studies demonstrate Adaboost's advantages in genomic prediction, particularly in handling complex non-linear interactions within high-dimensional data. It has been shown to effectively improve prediction accuracy for various complex traits compared to traditional linear models [77, 78]. The Adaboost.RT regression model can be written as:

14 $$\begin{array}{c}f\left(\boldsymbol{x}\right)=\sum_{a=1}^{M}\left(log\frac{1}{{\varepsilon}_{a}}\right){f}_{a}\left(x\right)/\sum_{a=1}^{M}\left(log\frac{1}{{\varepsilon}_{a}}\right),\end{array}$$

where $f\left(\boldsymbol{x}\right)$ represents the final predicted value; ${f}_{a}\left(\boldsymbol{x}\right)$ denotes the prediction value of the a-th weak learner; ${\varepsilon}_{a}$ is the error rate of ${f}_{a}\left(\boldsymbol{x}\right)$.

Gradient boosting machines (GBM) employ gradient descent error minimization with boosting techniques to improve the overall goal of more accurately estimating the target response by iteratively fitting new models [79]. Boosting is commonly used in ML to combine weak predictor models, or weak learners, into a stronger model. The final predictive model is expressed by the following formula:

15 $$\begin{array}{c}\boldsymbol{y}=\mu +\sum_{i=1}^{M}v\times {h}_{i}\left(\boldsymbol{y}; \boldsymbol{X}\right)+\boldsymbol{e},\end{array}$$

where $\boldsymbol{y}$ represents the phenotypes and $\boldsymbol{X}$ the genotypes. The model starts with an initial prediction μ (the population mean). At each iteration i, rather than fitting $\boldsymbol{y}$ directly, GBM trains a new weak learner ${h}_{i}$ to fit the negative gradient of the loss function with respect to the prediction of the previous model. The contribution of ${h}_{i}$ is scaled by a shrinkage factor v (learning rate) and added to the cumulative model. This process is repeated M times, with each iteration incrementally reducing the overall loss, until the final residual $\boldsymbol{e}$ is minimized.

In addition to Adaboost and GBM, currently popular algorithms such as gradient boosting decision trees (GBDT) [80] and extreme gradient boosting (XGBoost) [81] also fall under the category of boosting algorithms. These advanced boosting frameworks typically incorporate regularization terms and second-order gradient information to enhance generalization. However, their performance is highly dependent on the configuration of hyperparameters, making rigorous hyperparameter tuning essential for achieving optimal predictive accuracy across different traits.

### Stacking

Stacking employs a hierarchical framework where multiple heterogeneous base learners are trained in the first layer, and their outputs serve as training data for a second-layer meta-model (Fig. 4c) [70]. Unlike bagging or boosting, stacking aims to leverage the diverse strengths of different algorithms to optimize predictive performance.

### Random forest

The random forest (RF), an ensemble learning algorithm proposed by Breiman [82], constructs a "forest" by combining the bagging method with multiple DTs. The RF regression model aggregates the predictions from individual base learners to form a robust final output, expressed by the following equation:

16 $$\begin{array}{c}f\left(\boldsymbol{x}\right)=\frac{1}{B}\sum_{b=1}^{B}{t}_{b}\left({\Psi}_{b}\left(y; \boldsymbol{x}\right)\right),\end{array}$$

where $f\left(\boldsymbol{x}\right)$ represents the predicted value from the RF regression; the predictor variable ${t}_{b}\left({\Psi}_{b}\left(y; \boldsymbol{x}\right)\right)$ is constructed using the bootstrap sample of data ${\Psi}_{b}\left(y; \boldsymbol{x}\right)$ during the b-th iteration of the DT; and B denotes the number of DTs included in the RF. By aggregating multiple weak learners through a voting or averaging mechanism, RF effectively forms a strong learner capable of processing high-dimensional biological datasets [83].

In GS, RF exhibits exceptional robustness and effectively mitigates overfitting, particularly when hyperparameters such as tree depth are appropriately tuned. However, it is important to acknowledge that RF shares the limitations of bagging regarding unbalanced data. The random subsetting process can result in bootstrap samples that lack sufficient representation of minority classes, potentially biasing the model. Therefore, applying class weights or stratified bootstrapping is often recommended to ensure fair learning across all classes. This robustness is further enhanced by the stochastic nature of its construction process, which involves both random sampling and random feature selection. Its predictive performance frequently surpasses other ML algorithms, particularly in handling high-dimensional data and modeling non-linear patterns inherent in genomic datasets [84, 85]. For instance, Wang et al. [77] demonstrated that RF and GBDT provided significant accuracy gains over traditional models for reproductive traits such as litter weight and piglets born alive, noting that precise hyperparameter tuning further boosted performance. Despite its advantages, the computational cost of RF training grows significantly with the increasing depth and number of nodes; however, this challenge is effectively mitigated by the algorithm's high potential for parallelization. Modern RF implementations, particularly when combined with feature selection tools like the Boruta algorithm, can significantly boost computational efficiency. Moreover, substantial training datasets are required to achieve stable and satisfactory predictive performance in complex animal breeding populations [86].

### Kernel-based algorithms

#### Support vector machines

The support vector machines (SVM), a non-parametric supervised learning method proposed by Cortes and Vapnik [87], demonstrate powerful adaptability for complex biological datasets [88, 89]. While SVM performs linear classification on separable data, its true strength lies in processing non-linearly separable data through kernel functions. These functions map the original input space into a high-dimensional feature space where data becomes linearly separable. This "kernel trick" enables the efficient computation of inner products in high-dimensional space directly within the original input space, significantly reducing computational complexity and memory usage [90, 91]. The SVM model is expressed as follows:

17 $$\begin{array}{c}f\left(\boldsymbol{x}\right)={\beta}_{0}+h{\left(\boldsymbol{x}\right)}^{T},\end{array}$$

where $f\left(\boldsymbol{x}\right)$ denotes the prediction function, $\boldsymbol{\beta}$ represents the weight vector, ${\beta}_{0}$ is the intercept term, and $h{\left(\boldsymbol{x}\right)}^{T}$ denotes the kernel function. The most commonly used kernel functions include: the linear kernel: $K\left(\boldsymbol{x}_{i},\boldsymbol{x}_{j}\right)=\boldsymbol{x}_{i}{\prime}\boldsymbol{x}_{j}$, the polynomial kernel: $K\left(\boldsymbol{x}_{i},\boldsymbol{x}_{j}\right)={\left(\gamma \boldsymbol{x}_{i}{\prime}\boldsymbol{x}_{j}+r\right)}^{d}$, the Gaussian kernel: $K\left(\boldsymbol{x}_{i}, \boldsymbol{x}_{j}\right)={exp}\left(-\gamma {\Vert \boldsymbol{x}_{i}\boldsymbol-{x}_{j}\Vert }^{2}\right)$, and the sigmoid kernel: $K\left(\boldsymbol{x}_{i},\boldsymbol{x}_{j}\right)={tan}h\left(\gamma \boldsymbol{x}_{i}{\prime}\boldsymbol{x}_{j}+r\right)$ [92].

In the context of GS, this mechanism is crucial as it transforms genomic prediction from linear to nonlinear models. Compared to traditional linear methods, this approach offers greater flexibility in capturing non-additive genetic effects across the entire genome, thereby enhancing the prediction accuracy of GEBVs. While SVM shares principles with kernel ridge regression (KRR), they differ in their assumptions; specifically, unlike SVM, KRR typically assumes that only certain feature data significantly influence breeding value estimation [93]. Existing research has demonstrated the potential of SVM methods in GS [94]. Despite its potential, SVM faces challenges with large datasets, where solving the quadratic programming problem becomes computationally intensive [95]. Furthermore, direct genotype input can be challenging in populations with low-density SNP data. To address these issues, Long et al. [96] proposed replacing raw genotype data with a genomic relationship matrix as the input kernel. This optimization transforms the feature-based SVM into a relationship-based model, which not only enhances computational efficiency but also helps prevent model overfitting, especially when dealing with low-density SNP data. Recent empirical evidence further underscores the robustness of SVM in livestock breeding. Cross-breed evaluations in beef and dairy cattle have demonstrated that SVM-based regression can achieve superior accuracy compared to GBLUP by effectively capturing complex genetic architectures [56]. In addition, in the context of poultry breeding, a recent study on yellow-feathered broilers has shown that support vector regression (SVR) models can significantly outperform traditional linear methods like GBLUP in predicting specific carcass traits, including half-eviscerated and eviscerated weights [67]. Regarding computational scalability, recent large-scale studies in swine populations validate that utilizing a genomic relationship matrix directly as the kernel input serves as an effective computational strategy. This approach significantly mitigates the computational intensity typically associated with SVM training while maintaining high predictive stability even with low-density genotype data [97].

#### Reproducing kernel Hilbert space

Reproducing kernel Hilbert space (RKHS) theory offers a powerful framework for statistical learning, primarily addressing three categories of problems. First, the subspace problem in function spaces [98], where mapping the problem space to a higher-dimensional space can make the problem easier to solve when defined precisely on an RKHS subspace. Second, the kernel-based embedding of positive semi-definite functions [99], which allows SVMs and other methods to transform nonlinear problems into linear ones via the kernel trick. A classic example is Parzen's [100] work linking RKHS to stochastic processes via covariance kernels. Third, geometric embeddings [101], where RKHS preserves distance-preserving transformations. These mathematical properties provide the foundation for mapping complex biological data into high-dimensional feature spaces. Building on these theoretical strengths, Gianola et al. [102] introduced RKHS to genomic prediction as a typical semi-parametric method. RKHS offers a more general covariance structure than GBLUP, with pedigree-based BLUP and MAS models being special cases of the RKHS framework [103]. Numerous studies have demonstrated that RKHS outperforms traditional parametric models in genome-wide selection [104–106]. A critical factor in RKHS specification is the choice of the reproducing kernel; multi-kernel models are particularly beneficial for capturing complex genetic architectures and simplifying kernel selection [107].

#### Other machine learning approaches

Beyond mainstream ML methods, several other approaches have been explored for genomic prediction, albeit less frequently. The k-nearest neighbors (KNN), a non-parametric method often used for classification and regression, estimates phenotypic values ($\widehat{{y}_{j}}$) based on the genomic proximity of the K nearest neighbors:

18 $$\begin{array}{c}\widehat{{y}_{j}}=\frac{1}{K}\sum_{k=1}^{K}{y}_{k},\end{array}$$

where $\widehat{{y}_{j}}$ is the predicted phenotype of individual j; K is the number of nearest neighbors; and ${y}_{k}$ is the observed phenotype of the k-th nearest neighbor found in the training set. The proximity of neighbors is defined by the genomic distance between individuals. The Euclidean distance $d\left(\boldsymbol{x}_{i},\boldsymbol{x}_{j}\right)$ between two individuals, i and j, is calculated using the formula shown below:

19 $$\begin{array}{c}d\left(\boldsymbol{x}_{i},\boldsymbol{x}_{j}\right)=\sqrt{\sum_{p=1}^{P}{\left(\boldsymbol{x}_{pi}\boldsymbol{-{x}}_{pj}\right)}^{2}},\end{array}$$

where P is the total number of SNP markers; $\boldsymbol{x}_{pi}$ and $\boldsymbol{x}_{pj}$ represent the genotype codes for the p-th SNP marker of individuals i and j, respectively. The prediction is derived from the K nearest individuals with the smallest distance values. When determining the K value, a guided search method (such as cross-validation) can be employed to test different K values, ultimately selecting the K value that minimizes the mean squared error (MSE) between the actual and predicted values.

In some studies, KNN-based genomic prediction accuracy in dairy cows [108] was lower than that of other classical and ML models. Similarly, naive Bayes (NB), a probabilistic classification algorithm, has also been employed in ML applications. It exhibits high predictive capability, though this varies depending on conditions and environments. The NB classification model has been successfully utilized to identify SNPs associated with mortality in chickens [109, 110].

NB demonstrated superior performance in predicting survival traits in Holstein cattle compared to RF [111], yet showed limitations in horse breed prediction relative to specialized Bayesian methods [112]. Other instance-based algorithms, such as IB1 and IB5, have also been tested but generally fall short of the predictive benchmarks set by Bayesian linear procedures [113]. Beyond these conventional approaches, advanced non-linear models have also been developed to address environmental complexity. The kernelized Bayesian matrix factorization (KBMF) algorithm has proven effective in capturing genotype-by-environment interaction (G × E), outperforming standard BLUP by integrating weather forecasts into breeding value estimations [114]. Originally demonstrated in crops, these kernel-based strategies are gaining traction in animal breeding for traits sensitive to management and climate, such as those in porcine populations [115].

### Integration of multi-omics data using machine learning in genomic selection

In addition to the aforementioned ML methods for genomic prediction, the current frontier of genomic prediction lies in the integration of multi-omics data to move beyond purely additive genetic models. It is crucial to acknowledge the "no free lunch" theorem in ML, which posits that no single algorithm universally outperforms others across all possible problems. The efficacy of a method is inherently tied to the specific data structure, the underlying genetic architecture of the trait, and the modeling assumptions. Early breakthroughs like multilayered LASSO pioneered the interactive learning of genetic, transcriptional, and metabolomic data in plants [116]. Similar multi-omics-guided frameworks have since been extended to animal and model organism breeding. These examples, while demonstrating the potential of integrating diverse data layers, also highlight that performance gains are context-dependent. For instance, Ye et al. [117] utilized whole-genome sequencing combined with gene expression data in the *Drosophila* Genetic Reference Panel. They preselected SNPs via expression QTL (eQTL) mapping of significant genes identified from transcriptome-wide association studies and incorporated these functionally informed SNPs into both GBLUP and genomic feature BLUP models. This approach resulted in substantially improved prediction accuracy for specific complex traits, underscoring the importance of tailoring the analytical approach to the biological question at hand.

Additionally, innovative kernel-based models such as transcriptome BLUP, multi-omics BLUP, multi-omics single-step BLUP, weighted multi-omics single-step BLUP, and fusion similarity BLUP have been introduced [118, 119]. These methods, particularly weighted multi-omics single-step BLUP, optimally integrate intermediate omics layers (e.g., transcriptomics) into the genomic relationship matrix. By capturing regulatory and functional variants that standard GBLUP misses, these fusion similarity approaches have yielded accuracy gains of 3.37%–4.18% in beef cattle across diverse genetic architectures [119]. Collectively, these studies underscore that the strategic incorporation of functional omics data, rather than simple feature expansion, is pivotal for the next generation of GS programs.

Parallel to these matrix-based approaches, Bayesian frameworks have also been actively developed to directly or indirectly integrate other omics layers. Instead of constructing multiple relationship matrices, these methods typically utilize multi-omics data to inform prior distributions. For instance, advanced models like BayesRC and BayesRCO indirectly integrate these layers by classifying SNPs into distinct functional categories based on omics annotations and applying differential shrinkage to biologically active regions. This Bayesian multi-omics integration effectively prioritizes causal variants, thereby enhancing both prediction accuracy and biological interpretability compared to BLUP-based methods.

## Deep learning methods in genomic selection

In genomic prediction, it is a universally recognized principle across all statistical and computational frameworks that model performance is highly task-dependent, with no single algorithm offering a universal solution. While classical ML algorithms have been extensively applied to genomic prediction, their capacity to improve accuracy is often constrained by the high dimensionality of genomic data and the computational intensity inherent in training. As a sophisticated evolution of ML, DL has emerged as a powerful alternative. While its success, like all genomic prediction methods, fundamentally relies on the availability of massive datasets, DL is uniquely characterized by its ability to construct multi-layered architectures that autonomously extract complex, non-linear features [120]. At the core of DL are artificial neural networks (ANN), which draw structural inspiration from the human brain's interconnected biological neurons [121]. An ANN typically comprises an input layer, multiple hidden layers, and an output layer. Within this hierarchy, each node or "neuron" receives input through learnable weights and activation thresholds [122]. The refinement of these parameters is governed by the Backpropagation algorithm, which minimizes prediction error by propagating error backward from the output to the preceding layers—a mechanism essential for training deep architectures [122].

In the context of GS, DL models represent the current state-of-the-art for functional prediction, particularly for complex quantitative traits. Their superior performance stems from an innate capacity for self-learning and the modeling of intricate, non-linear biological relationships [123, 124]. Recent advances have also seen the rise of explainable AI within regulatory genomics, aiming to decode the "black box" of DL models [125]. The following sections provide a detailed review of the DL architectures most extensively utilized in the current GS landscape.

### Multilayer perceptron

The multilayer perceptron (MLP), a fundamental class of feedforward neural networks, was first introduced to the field of genomic prediction by Okut et al. [126] to predict body weight index in mice. Structurally, an MLP consists of an input layer, one or more hidden layers, and an output layer. Each layer comprises processing units termed neurons, which are interconnected through a system of weights (w) that represent the strength of the connections [127, 128]. In a typical MLP, each neuron in a hidden layer receives inputs from all neurons in the preceding layer, computes a weighted sum, and then applies a non-linear activation function (σ) to generate an output. From a biological perspective, the step function (either 0 or 1) most closely mirrors the excitation-inhibition mechanism of biological neurons. However, due to its non-differentiability, modern practical applications predominantly utilize the sigmoid or ReLU functions to facilitate gradient-based optimization. Figure 4d presents a diagram of a three-layer MLP with four input layer units, five hidden layer units, and one output layer unit. Formally, an MLP can be defined as a universal function approximator. The general mapping from genomic markers to phenotypes is expressed as:

20 $$\begin{array}{c}\boldsymbol{y}=f\left(\boldsymbol{X}; \theta \right),\end{array}$$

where $\boldsymbol{X}$ is the n×p input matrix representing p genomic markers for n individuals, $\boldsymbol{y}$ is the n×1 output vector of phenotypes, and θ denotes the set of all learnable parameters (weights and biases). The goal of $f\left(\bullet \right)$ is to learn an optimal non-linear mapping from $\boldsymbol{X}$ to $\boldsymbol{y}$. The final model output is a linear combination of the non-linear transformations from the terminal hidden layer:

21 $$\begin{array}{c}\boldsymbol{y}=f\left(\boldsymbol{X}; \theta , \boldsymbol{W}, \boldsymbol{b}\right)=\phi {\left(\boldsymbol{X}; \theta \right)}^{T}\boldsymbol{W}+\boldsymbol{b},\end{array}$$

where $\phi \left(\boldsymbol{X}; \theta \right)$ represents the final hidden representation after $\boldsymbol{X}$ has passed through all hidden layers (parameterized by θ and their non-linear transformations. $\boldsymbol{W}$ and $\boldsymbol{b}$ are the weights and biases of the output layer, respectively. For a single-layer MLP, this process is simplified to:

22 $$\begin{array}{c}\widehat{\boldsymbol{y}}=\sigma \left(\boldsymbol{X{W}}_{1}+\boldsymbol{b}\right)\boldsymbol{W}_{2},\end{array}$$

where $\boldsymbol{W}_{1}$ and $\boldsymbol{b}$ connect the input layer to the hidden units, and $\boldsymbol{W}_{2}$ connects the hidden layer to the output. σ is the non-linear activation function applied to the hidden layer. To optimize the learnable parameters, the network minimizes a loss function representing the discrepancy between predicted and observed values [129]. For regression tasks in GS, the MSE is the standard objective function:

23 $$\begin{array}{c}L\left(\boldsymbol{y}, \widehat{\boldsymbol{y}}\right)=\frac{1}{2n}\sum_{i=1}^{n}\| {y}_{i}-\widehat{{y}_{i}}{\| }_{2}^{2},\end{array}$$

where $L\left(\boldsymbol{y}, \widehat{\boldsymbol{y}}\right)$ is the total loss. n is the total number of individuals (observations) in the training set. ${y}_{i}$ is the true observed phenotype for individual i; and $\widehat{{y}_{i}}$ is the predicted phenotype for individual i. $\| \bullet {\| }_{2}^{2}$ denotes the Euclidean squared norm, which calculates the squared difference between ${y}_{i}$ and $\widehat{{y}_{i}}$. The weights are iteratively adjusted to minimize L, though the objective function may exhibit multiple local minima, potentially leading to convergence at sub-optimal solutions.

Despite their theoretical promise, empirical results for ANNs in genomic prediction remain mixed; for instance, while some authors [130] reported improved prediction accuracy for complex traits in Nellore cattle, whereas others found that gradient boosting and SVM algorithms outperformed ANNs [71, 131]. Still other researchers [132, 133] noted that although ANN performance varied across traits, it generally did not surpass that of classical or other non-parametric models. Despite its theoretical promise, the ANN approach faces several practical challenges: it typically requires very large datasets for effective training, is prone to overfitting, and depends on numerous hyperparameters whose optimal values can be difficult to identify [134, 135]. Consequently, ongoing research focuses on improving hyperparameter optimization [136, 137]. For instance, applying "differential evolution" as a population-based evolutionary heuristic can serve as an effective search method within the arbitrarily complex hyperparameter space of DL models [136]. Furthermore, the objective function may exhibit multiple local optima, and some algorithms may converge to suboptimal solutions where parameter values yield poor predictive capability. To mitigate the issues of regularization and uncertainty, Bayesian neural networks (BNNs) have emerged as a robust variant. By assuming a joint prior distribution over the weights and inferring a posterior distribution from the data, BNNs provides a probabilistic framework for training. This approach allows for weight sampling during forward passes and establishes a theoretical link to Gaussian processes, showing significant potential in animal breeding applications [128, 138–140].

### Convolutional neural networks

Convolutional neural networks (CNNs) are a class of feedforward neural networks inspired by cognitive neuroscience. Unlike MLPs, where hidden layers are exclusively fully connected, CNNs architectures integrate convolutional layers, pooling layers, and fully connected layers [71, 134, 141]. This specialized structure allows CNN to capture local spatial correlations and hierarchical patterns within input data.

The convolutional layer serves as the primary feature extractor. By sliding a trainable convolution kernel (or filter) over the input, which in GS studies typically consists of one-dimensional SNP sequences, the model performs multiply-accumulate operations to generate feature maps [142]. The core operation of the convolutional layer is defined as follows:

24 $$\begin{array}{c}{g}_{l}\left(x\right)=\phi \left(\sum_{{l}{\prime}=1}^{p}\left({f}_{{l}{\prime}}*{K}_{{l}{\prime}, l}\right)\left(x\right)\right).\end{array}$$

In this formula, we define the mapping from a p-dimensional input $f(x)=\left(f_{1}(x),\cdots, f_{p}(x)\right)$ to a Q-dimensional output ${g({x})=({g}}_{{1}}{({x}),\cdots , {g}}_{{q}}{({x}))}$. First, the ${l}{\prime}$-th input feature map ${f}_{{l}{\prime}}$ is convolved (denoted by ∗) with a trainable kernel ${K}_{{l}{\prime}, l}$. The kernel ${K}_{{l}{\prime}, l}$ represents the specific filter connecting the ${l}{\prime}$-th input channel to the l-th output channel. Subsequently, the results from all p input channels are aggregated using the summation operator ∑. This step accumulates information from all input dimensions to generate the pre-activation value for the l-th output channel. Finally, this aggregated value is passed through a non-linear activation function ϕ to obtain the final l-th output feature map, ${g}_{l}\left(\boldsymbol{x}\right)$.

To further condense the extracted features and enhance computational efficiency, a pooling layer is typically introduced. Common methods include max pooling and average pooling, which reduce the spatial dimensions of feature maps while maintaining robustness to positional variations:

25 $$\begin{array}{c}{g}_{l}\left(\boldsymbol{x}\right)=P\left({f}_{l}\left(\boldsymbol{x}{\prime}\right):\boldsymbol{x}{\prime}\in\boldsymbol{V}\left(\boldsymbol{x}\right)\right).\end{array}$$

This formula describes a feature aggregation process: $\boldsymbol{V}{(}\boldsymbol{x}{)}$ defines a non-overlapping or overlapping pooling window centered at position $\boldsymbol{x}$. P is the pooling function, which aggregates all input values ${f}_{l}\left(\boldsymbol{x}{\prime}\right)$ within the $V\left(\boldsymbol{x}\right)$ neighborhood into a single output value. ${g}_{l}\left(\boldsymbol{x}\right)$ is the resulting summarized feature map obtained after pooling the l-th feature map ${f}_{l}$. Finally, one or more fully connected layers integrate these high-level features to perform phenotypic prediction tasks, including regression or classification [143].

In the context of GS, CNN-based models such as DeepGS [144] and ResGS [145] have demonstrated significant potential. While still largely exploratory, CNNs have shown superior performance in predicting traits like litter size and accommodating large non-additive genetic variances [71, 141]. This advantage is largely attributed to their ability to automatically extract local features and capture high-order epistatic interactions among markers through convolutional filters, which are crucial for complex traits governed by non-additive effects. Beyond single-omic prediction, CNNs are increasingly utilized to integrate multi-omics data. For instance, Fu et al. [146] developed a CNN model to prioritize candidate genes by merging diverse omics information, supported by the ISwine knowledge base. Another prominent DL algorithm is the recurrent neural network (RNN) [135]. Due to their inherent architecture, RNNs are highly suitable for modeling spatio-temporal structures and processing sequential information, finding wide application in fields such as time series prediction, natural language processing, and speech recognition [147]; however, their use in GS remains limited. Specifically, the training process for RNNs, particularly due to their complex feedback mechanisms, typically demands significant computational resources. In practical applications, the use of RNNs still faces several challenges [148]. To clarify the hyperparameter settings and optimization strategies for the models used in this study, we summarize them in Table 2.

**Table 2:** Summary of key hyperparameters, optimization methods, and tuning tools for major ML and DL models used in GS

Category	Representative methods	Key hyperparameters	Common optimization methods	Hyperparameter tuning tools
Tree-based ensemble	GBM (e.g., XGBoost)	Learning rate, number of trees ($n\_estimators$), max depth of trees, subsample ratio, min child weight	Grid search, random search, Bayesian optimization	Common hyperparameter optimization libraries: Optuna, Hyperopt, and scikit-optimize; hyperparameter tuning using AutoML libraries: Auto-Sklearn, H2O AutoML, and Auto-PyTorch; Algorithm-based hyperparameter tuning: Bayesian optimization, HyperBand, and genetic algorithms
	RF	Number of trees ($n\_estimators$), max depth of trees, number of features (${m}_{try}$), min samples per leaf	Grid search, random search, out-of-bag error estimation	
Kernel-based	SVM/SVR	Regularization parameter (C), kernel coefficient (γ), kernel type, epsilon (ϵ)	Grid search, random search, cross-validation	
	RKHS/KRR	Kernel function, bandwidth (h), regularization parameter (λ)	Grid search, cross-validation	
	KNN	Number of neighbors (k), distance metric (Euclidean, Manhattan, etc.), weight function	Grid search, cross-validation	
DL	ANN (MLP)	Learning rate, number of hidden layers/neurons, activation function, batch size, dropout rate, regularization (${L}_{1}$/${L}_{2}$)	Grid search, random search, Bayesian optimization, differential evolution	
	CNN	Number of convolutional layers/filters, kernel size, stride, pooling type, padding, learning rate	Grid search, random search, Bayesian optimization	

### Recent empirical applications and comparative studies

This section synthesizes recent empirical studies comparing classical parametric models with non-parametric ML and DL methods in GS. To ensure a comprehensive review, we conducted a literature search using keywords such as "genomic selection", "machine learning", "deep learning", "genomic prediction", and "animal breeding" across databases including PubMed, Web of Science, and Google Scholar. The inclusion criteria focused on peer-reviewed studies published in English that explicitly compared ML/DL methods with traditional statistical methods in animal breeding contexts. We prioritized recent studies (mainly within the last 5–10 years) to capture the latest advancements, while also including seminal works essential for the theoretical context. By evaluating the performance of these selected studies across major livestock species, we focus on the prediction accuracy of complex traits. Table 3 summarizes these key investigations from 2018 to 2026, illustrating the relative strengths and current trends in genomic prediction for animal breeding programs.

**Table 3:** Summary of empirical comparisons (2018–2026) between parametric models and ML methods in GS across major livestock species

Species	Year	References	No. of animals	No. of SNPs	Traits	Traditional/ML methods	Key findings
Pig (real and simulated data)	2018	Waldmann [139]	3,226 (simulated), 3,141 (real)	9,723 (simulated), 50,276 (real)	Quantitative trait	GBLUP, BL, ABNN	ABNN outperformed GBLUP and BL in prediction accuracy
Pig (real and simulated data)	2020	Waldmann et al. [141]	3,226 (simulated), 808 (real)	9,723 (simulated), 50,174 (real)	Quantitative trait	GBLUP, LASSO, CNN	CNN reduced prediction error by > 25% in simulated data and ~3% in real pig data compared to GBLUP and LASSO
Chinese Yorkshire pigs	2022	Wang et al. [86]	2,566	44,922	Total number of piglets born and number of piglets born alive	GBLUP, ssGBLUP, BayesHE, SVR, KRR, RF, Adaboost.R2	ML methods significantly outperformed traditional methods, and hyperparameter tuning substantially improved ML performance
Pigs (PIC commercial dataset + real dataset from Chifeng national pig nucleus herd, North China)	2023	Xiang et al. [85]	2,691–3,161 (PIC), 4,307–6,211 (Chifeng)	41,670 (PIC), 34,604 (Chifeng)	T1, T2, T3, T4, T5, average daily gain, and total number of piglets born	GBLUP, RF, SVM, XGBoost, CNN	Non-linear ML approaches demonstrated feasibility and reliability for genomic prediction and genetic site screening in pigs, especially capturing complex/non-linear relationships
Rongchang pigs (Chinese local breed)	2025	Wu et al. [149]	485	22,686 (chip), 3,472,412 (imputed)	Backfat thickness, loin and thoracic height, and girth circumference	GBLUP, Bayesian, KRR, SVR, RF, GBDT, LightGBM, Adaboost	ML methods outperformed traditional methods by 6.6–8.1% in prediction accuracy
Pigs (multiple)	2025	Su et al. [150]	1,187–4,146 (5 datasets)	32,909–52,843 (5 datasets)	Reproductive and growth traits; PIC traits are hidden	GBLUP, LASSO, SVR, KRR-rbf, KRR-cos, KRR-sig, stacking, Adaboost, RF, ENET, CNN, MLP	GBLUP showed strong performance; stacking, SVR, and KRR-rbf also achieved high prediction accuracy and stability
Holstein dairy cattle (real and simulated data)	2020	Abdollahi et al. [71]	3,226 (simulated), 3,141 (real)	9,723 (simulated), 50,276 (real)	Sire conception rate	GBLUP, BayesB, RF, GBM, MLP, CNN	GBM outperformed DL and traditional methods in the real bull fertility dataset, while DL showed limited advantages
Hanwoo cattle	2021	Srivastava et al. [151]	7,324	45,624	Carcass weight, marbling score, backfat thickness, and eye muscle area	GBLUP, RF, XGBoost, SVM	XGBoost outperformed for carcass weight and marbling score, whereas GBLUP was best for backfat thickness and eye muscle area; however, GBLUP showed the lowest MSE for all traits
Cattle (Holstein Friesian)	2022	Nazzicari et al. [152]	1,033	26,503	Simulated complex traits: additivity, dominance, and epistasis	Stacked kinship CNN, GBLUP-A, GBLUP-optim	GBLUP outperformed CNN: CNN showed lower root MSE but also lower Pearson correlation and normalized discounted cumulative gain compared to GBLUP-optim. GBLUP remains a strong benchmark for additive traits
Chinese Holstein dairy cattle	2024	Wang et al. [153]	5,962–6,558	45,944	Seven traits: milk production traits, type traits, and one health trait	GBLUP, BayesB, BayesC*π* RF, BANNs	BANNs framework (especially BANN_100kb) outperformed GBLUP, RF, BayesB, and BayesC*π* across all seven traits
North American Holstein dairy cattle	2024	Pedrosa et al. [154]	4,511	57,600	Behavioral traits from automatic milking systems	GBLUP, BL, MLP, CNN	DL methods (MLP/CNN) showed slightly higher accuracies than GBLUP, but the results may not be sufficient to justify their use over traditional methods due to higher computational demand
Nellore beef cattle	2024	Mota et al. [155]	1,024	305,128	Feed efficiency-related traits	STGBLUP, MTGBLUP, BayesA, BayesB, BayesC, BRR, BL, MLNN, SVR	SVR and MTGBLUP outperformed MLNN, STGBLUP, and all Bayesian regression methods
Composite beef cattle	2024	Hay [156]	4,680	40,533	Birth weight, weaning weight, and yearling weight	GBLUP, BayesA, BayesB, SVM, CNN, MLP, RF	GBLUP showed robust performance, though RF outperformed it for weaning weight
Holstein dairy cattle	2026	Schwarz et al. [157]	7,413	45,161	Endometritis, retained placenta, stillbirth maternal, and ovary cycle disturbances	GWAS (mixed linear model), RF	RF complements GWAS: identified non-additive genetic architectures and epistatic effects missed by GWAS; top RF markers showed chromosomal clustering different from GWAS signals
Inner Mongolian cashmere goats	2025	Liu et al. [158]	2,256	50,728	Fiber length, cashmere diameter, and cashmere production	GBLUP, RF, XGBoost, GBDT, LightGBM	LightGBM, RF, and GBDT provided higher genomic prediction accuracy than traditional GBLUP for cashmere traits in cashmere goats
Yellow-feathered broilers	2025	Liu et al. [67]	989–1,347	16,962–31,934	Eight traits: laying, growth, and carcass traits (highlighted half-eviscerated weight and eviscerated weight)	GBLUP, BayesA, BayesB, BayesC, BL, BRR, SVR, RF, GBDT, XGBoost, LightGBM, KRR, MLP	Traditional methods (GBLUP and Bayesian) outperformed ML methods in 5 out of 8 traits, whereas ML methods (especially SVR and RF) showed significantly higher accuracy for carcass traits (highlighted half-eviscerated weight and eviscerated weight)
Yellow-feathered broilers	2026	Chai et al. [159]	834	500–100,000	Eight traits: egg production and quality traits	GBLUP, MLP, RF	RF outperformed for low-heritability traits (e.g., egg number, shell traits), while GBLUP was better for high-heritability egg weight traits
Simulated livestock population	2025	Yang et al. [160]	12,500 (training), 500 (testing)	55,043	Quantitative trait	GBLUP, 2GBLUP, weighted 2GBLUP, RF, SVR	QTL integration benefits GBLUP/SVR but not RF, possibly due to data structure or RF's unsuitability for this genomic prediction scenario

All abbreviations used in this table are defined in the 'Abbreviations' section

As summarized in Table 3, empirical studies from 2018 to 2026 have increasingly explored ML and DL methods in GS, frequently benchmarking them against traditional linear models like GBLUP, ssGBLUP, and Bayesian regressions. Results illustrate that ML approaches often outperform traditional parametric models, particularly for traits characterized by complex or non-additive genetic architectures. However, performance varies depending on trait heritability, population size, and validation design. Most investigations focus on pigs and dairy or beef cattle. However, emerging applications can also be seen in goats and poultry. The evaluated traits cover a broad range: specifically, for cattle, these include milk production and behavioral traits. In pigs, the focus is on reproductive and growth traits. Other examples include cashmere production in goats, as well as egg production and carcass traits in broilers. Despite these advancements, the landscape of genomic prediction faces new hurdles. With the exponential surge in the number of whole-genome markers, achieving accurate predictions under high-dimensional and small-sample conditions remains a formidable challenge. While traditional methods like KNN and SVM have been widely adopted, limitations exist. Notably, recent studies by Ali et al. [161] on human genomic data demonstrate that the improved double-weighted k-nearest neighbor algorithm can more effectively filter out noise. By incorporating feature weighting and exponential distance weighting, it enhances classification accuracy, suggesting that integrating such weighted distance algorithms into animal GS models represents a promising direction for future breeding value predictions.

Overall, non-parametric ML and DL approaches frequently outperform traditional parametric models, particularly for complex or low-heritability traits involving non-additive effects or heterogeneous genetic architectures. In empirical studies, reported accuracy gains range from 3% to 10%, whereas these gains can reach up to 25%. Ensemble methods and kernel-based techniques demonstrate particular robustness and stability across scenarios. DL architectures excel when non-linear interactions or large datasets are present; however, they are sensitive to hyperparameter tuning and risk overfitting. In contrast, linear models like GBLUP remain competitive or superior for additive, high-heritability traits and smaller datasets, while also offering advantages in computational efficiency and interpretability. These trends underscore the potential of ML and DL to address limitations of classical frameworks in the big data era, yet they also highlight the critical need for larger reference populations and rigorous model validation.

## Conclusions and future perspectives

The statistical foundations of GS have undergone a remarkable evolution, beginning with pedigree-based linear mixed models that excel in predicting additive, high-heritability traits. The field then progressed to Bayesian regressions, which better accommodate heterogeneous marker effects and oligogenic architectures. Today, in the era of big data breeding, non-parametric ML and DL approaches are capable of modeling complex nonlinear interactions. Traditional parametric models remain computationally efficient and robust; consequently, they are widely implemented in routine livestock breeding programs, providing reliable predictions with interpretable assumptions. In contrast, ML and DL methods demonstrate increasing promise for low-heritability or non-additive traits, and empirical studies in cattle, pigs, poultry, and goats have shown clear accuracy gains with these advanced methods.

Nevertheless, no single method universally dominates; performance depends on trait genetic architecture, dataset size, population structure, and computational resources. Ensemble techniques and kernel-based methods often provide stable improvements with moderate demands, whereas DL architectures shine in large-scale, high-dimensional scenarios but require careful hyperparameter optimization to mitigate overfitting and enhance interpretability. Looking ahead, hybrid models combining parametric efficiency with non-parametric flexibility, alongside multi-omics integration and explainable AI, are poised to accelerate genetic progress in animal breeding further. These advances will support sustainable livestock production by enabling more precise selection for complex traits such as disease resistance, feed efficiency, and environmental resilience. Ultimately, judicious model selection, guided by empirical validation and breeding objectives, will maximize genomic gains in practical programs worldwide.

## Data Availability

No datasets were generated or analysed during the current study.

## Abbreviations

ABNN: Approximate Bayesian neural networks
Adaboost: Adaptive boosting
ANN: Artificial neural networks
BANNs: Biologically annotated neural networks
BayesHE: Bayesian horseshoe
BL: Bayesian Lasso
BLUP: Best linear unbiased prediction
BNN: Bayesian neural networks
BRR: Bayesian ridge regression
CART: Classification and regression tree
CNN: Convolutional neural networks
DL: Deep learning
DT: Decision tree
ENET: Elastic net
eQTL: Expression QTL
GBDT: Gradient boosting decision trees
GBLUP: Genomic BLUP
GBM: Gradient boosting machines
GEBV: Genomic estimated breeding values
G × E: Genotype-by-environment interaction
GS: Genomic selection
GWAS: Genome-wide association study
KBMF: Kernelized Bayesian matrix factorization
KNN: K-nearest neighbors
KRR: Kernel ridge regression
LD: Linkage disequilibrium
LightGBM: Light gradient boosting machine
MAS: Marker-assisted selection
MCMC: Markov chain Monte Carlo
MH: Metropolis-Hastings
ML: Machine learning
MLNN: Multi-layer neural network
MLP: Multilayer perceptron
MME: Mixed model equations
MSE: Mean squared error
MTGBLUP: Multi-trait GBLUP
NB: Naive Bayes
QTL: Quantitative trait locus
RF: Random forest
RKHS: Reproducing kernel Hilbert space
RNN: Recurrent neural network
RRBLUP: Ridge regression BLUP
SNP: Single nucleotide polymorphism
ssBR: Single-step Bayesian regression
ssGBLUP: Single-step GBLUP
STGBLUP: Single-trait GBLUP
SVM: Support vector machines
SVR: Support vector regression
TABLUP: Trait-specific marker derived relationship matrix BLUP
XGBoost: Extreme gradient boosting
